# Ethyl Glucuronide in Hair: A 10‐Year Overview

**DOI:** 10.1002/dta.70041

**Published:** 2026-02-18

**Authors:** Sara Casati, Alessandro Ravelli, Roberta F. Bergamaschi, Caterina Fabbri, Erika Palmisano, Andrea Ostinelli, Gabriella Roda, Giuseppe Facchi, Marica Orioli

**Affiliations:** ^1^ Laboratorio di Tossicologia Forense, Dipartimento di Scienze Biomediche, Chirurgiche ed Odontoiatriche Università Degli Studi di Milano Milan Italy; ^2^ Fondazione Ca' Granda IRCCS Ospedale Maggiore Policlinico Milan Italy; ^3^ Dipartimento di Scienze Farmaceutiche Università Degli Studi di Milano Milan Italy; ^4^ Dipartimento di Informatica Giovanni Degli Antoni Università Degli Studi di Milan Milan Italy; ^5^ Dipartimento di Scienze Biomediche, Chirurgiche ed Odontoiatriche Università Degli Studi di Milano Milan Italy

**Keywords:** addiction, alcohol, EtG, hair analysis, LC‐MS/MS

## Abstract

This 10‐year retrospective study evaluates hair ethyl glucuronide (EtG), a direct metabolite of ethanol, in a large Northern Italian cohort (*N* = 68,221 samples collected between 2013 and 2022). The analysis aimed to evaluate long‐term alcohol consumption through the EtG distribution in terms of age, gender, recidivism, applicants, sampling region, sampling length, and the impact of seasonality and the COVID‐19 pandemic. Hair EtG was determined by HPLC‐MS/MS, and samples were classified according to the Society of Hair Testing (SoHT) recommended cut‐offs: EtG ≤ 5 pg/mg (does not contradict abstinence), 5 < EtG < 30 pg/mg (repeated alcohol consumption), and EtG ≥ 30 pg/mg (chronic excessive alcohol consumption). A descriptive sensitivity analysis using SoHT cut‐offs was performed to allow international comparability. The percentage (%) of hair samples classified as repeated or chronic excessive alcohol consumption was 13.6% (*N* = 9,281) and 5.6% (*N* = 3,818), respectively. EtG values significantly varied with gender and age as well as referral context. We observed statistically significant differences in EtG concentrations recorded in head, chest, axillary, and pubic hair samples. Significantly higher EtG values were detected in 3‐cm proximal head hair versus the 3‐ to 6‐cm proximal segment as well as across seasons, with a higher concentration in colder months. Conversely, no measurable short‐term population impact of COVID‐19 was revealed by hair EtG, whereas a significant long‐term influence was highlighted. Overall, despite a large variability of EtG concentrations, this study provides robust evidence of the reliability of hair EtG testing and offers a comprehensive overview of long‐term alcohol consumption trends in a monitored Italian population.

## Graphical abstract

The present study builds on a unique collection of more than 68,000 hair EtG results collected in the Northen Italy between 2013 and 2022. We investigated trends in alcohol consumption in terms of age, gender, recidivism, applicants, sampling region, sampling length, across seasons, and the COVID‐19.

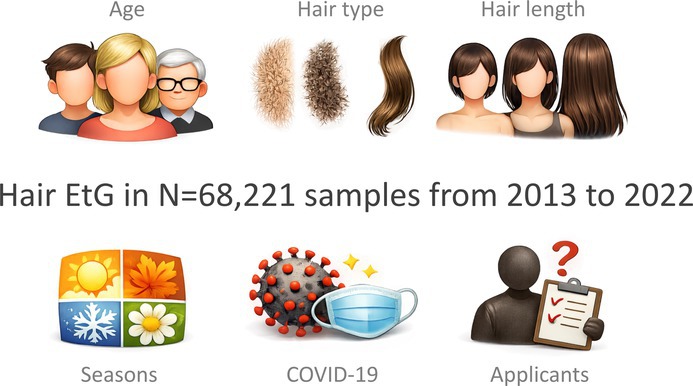

## 1 Introduction

Alcohol consumption is a major risk factor for global health, contributing to maternal and child morbidity, infectious and noncommunicable diseases, mental health disorders, injuries, and poisonings [1, 2, 3]. According to the World Health Organization (WHO), nearly 4.6% of the global burden of disease and injury is attributable to alcohol each year, with hazardous drinking responsible for an estimated 2.6 million deaths annually [1]. Effective monitoring of harmful alcohol use therefore requires objective diagnostic tools capable of distinguishing abstinent, moderate, and excessive drinkers. Available biomarkers include indirect indicators and direct metabolites of ethanol, with varying levels of reliability [4]. Among direct biomarkers, ethyl glucuronide (EtG) in hair has emerged over the past decade as one of the most sensitive and specific indicators of chronic alcohol consumption [5]. Hair EtG testing is now widely accepted in forensic and clinical toxicology, with applications in workplace monitoring, transplantation medicine, firearms licensing, driving license reinstatement, and postmortem investigation [6]. The WHO defines chronic excessive drinking as daily intake ≥ 60 g of pure alcohol over months or years, whereas the International Society of Hair Testing (SoHT) recommends cut‐off values of 30 pg/mg for excessive drinking and 5 pg/mg to support abstinence [7]. Standardized sampling from the posterior vertex region of the scalp is preferred, although body hair may be collected if scalp hair is unavailable. In such cases, the physiological differences in growth cycle and growth rate of non‐head hair (i.e., axillary and pubic hair) must be considered for the interpretation of the results [8]. The present study builds on a unique collection of more than 68,000 hair EtG results generated at the Laboratory of Forensic Toxicology of the University of Milan between 2013 and 2022. This large sample dataset, originated primarily from driving license regranting procedures, rehabilitation programs, and workplace testing, allows us to (1) investigate long‐term temporal trends in alcohol consumption within a Northern Italian population in terms of age, gender, recidivism, applicants, sampling region, and sampling length; (2) evaluate the role of seasonality; and (3) assess the short‐ and long‐term impact of the COVID‐19 pandemic on alcohol use. Taken together, these data provide an extensive overview of EtG hair concentrations in a Northern Italian cohort routinely monitored for chronic alcohol consumption.

## 2 Material and Methods

### 2.1 Datasets

The hair EtG analytical results used in the present study arose from samples collected over a 10‐year period (from January 2013 to December 2022, *N* = 68,221) from (1) subjects whose driving license was temporarily suspended for administrative/legal sanctions, (2) individuals constantly monitored due to their ongoing or past alcohol‐dependence conditions, (3) professional workers undergoing workplace testing, and (4) others (including private, transplantation medicine, and firearms licensing). Post‐mortem hair samples were excluded from the study. All the subjects involved in the study gave their written informed consent for the anonymous use of their samples for research purposes.

Sample classification into three drinking categories was performed using the following EtG cut‐off values, according to SoHT guidelines: EtG values ≤ 5 pg/mg do not contradict abstinence, values > 5 and < 30 pg/mg indicate repeated alcohol consumption, and values ≥ 30 pg/mg indicate chronic excessive alcohol consumption.

The dataset (EtG ≥ LOQ) was stratified into subgroups based on age, gender, recidivism, applicants, sampling region, sampling length, and seasonality. This study evaluates also both the short‐ and long‐term impact of the COVID‐19 pandemic and lockdown on the population's alcohol intake by measuring the hair EtG in samples collected with the appropriate time‐shift. The lockdown protocol started in Italy on March 8, 2020, and it finished at the end of May 2020. Considering that the hair growth rate is about 1 cm/month and, commonly, the analysis is performed on the proximal 3‐ to 6‐cm segment of head hair, hair samples collected from April 2020 to August 2020 were selected for the study on the short‐term impact of COVID‐19. Finally, hair samples were collected from February 2019 to February 2020 (pre‐pandemic period) and from March 2020 to February 2021 (pandemic and postpandemic periods) for a long‐term impact evaluation.

Information on cosmetic hair treatments (e.g., dyeing, bleaching, and perming) was not systematically available and could therefore not be included in the present analysis.

### 2.2 Hair Collection and LC‐MS/MS Analysis

Scalp hair was collected by cutting hair close to the skin in the occipital or vertex region of the head: only the proximal ≤ 6‐cm segment was collected. Body hair (chest, axillary, and pubic hair) was collected by cutting or shaving close to the skin. Hair EtG extraction and analysis were performed according to a previously published LC‐MS/MS in‐house method meeting the requirements of DIN EN ISO/IEC 17025 standards [9]. The analytical method and the instrumentation (Dionex UltiMate 3000 HPLC system coupled to a QTRAP 4000 triple quadrupole mass spectrometer) were consistently used without significant modifications throughout the entire 10‐year study period, as recently published [10]. Limits of detection (LOD) and quantification (LOQ) remained stable at 2 and 6.7 pg/mg, respectively. Routine maintenance and replacement of consumables were performed under our accredited quality system, ensuring method continuity and performance consistency. The laboratory also participated successfully and continuously in external proficiency testing (PT) for EtG in hair during the 10 years, with results consistently falling within acceptable ranges, thereby confirming the reliability of the presented data [10].

### 2.3 Statistics and Data Interpretation

The first phase of data analysis focused on evaluating the number of subjects, percentage frequencies, and relative differences across various categories of alcohol consumption (chronic and repeated alcohol users). In the second phase, Student's *t* test (*t* test) or an analysis of variance (ANOVA) was performed to assess whether the observed differences in terms of EtG concentration were statistically significant or could be attributed to random variability within the dataset. In all tests, the significance level was set at *p* < 0.05. The maximum plotted hair EtG concentration was 150 pg/mg in order to exclude extreme outliers.

### 2.4 Software

All data processing and statistical analyses were performed using Python 3.11 or Microsoft Excel (v.2510). Numerical operations and descriptive statistics were carried out with the NumPy (v1.26), pandas (v2.1), and Microsoft Excel (v. 2510) equipped with the Real Statistics (v9.7). Inferential analyses, including *t* tests and ANOVA, were conducted with the scikit‐learn (v1.3) package and Microsoft Excel (v. 2510) equipped with the Real Statistics (v9.7). Data visualization, such as box plots and distribution graphs, was produced with built‐in plotting tools from pandas, matplotlib (v3.8), and MATLAB (v.R2025b).

## 3 Results

### 3.1 General Overview

**FIGURE 1:**
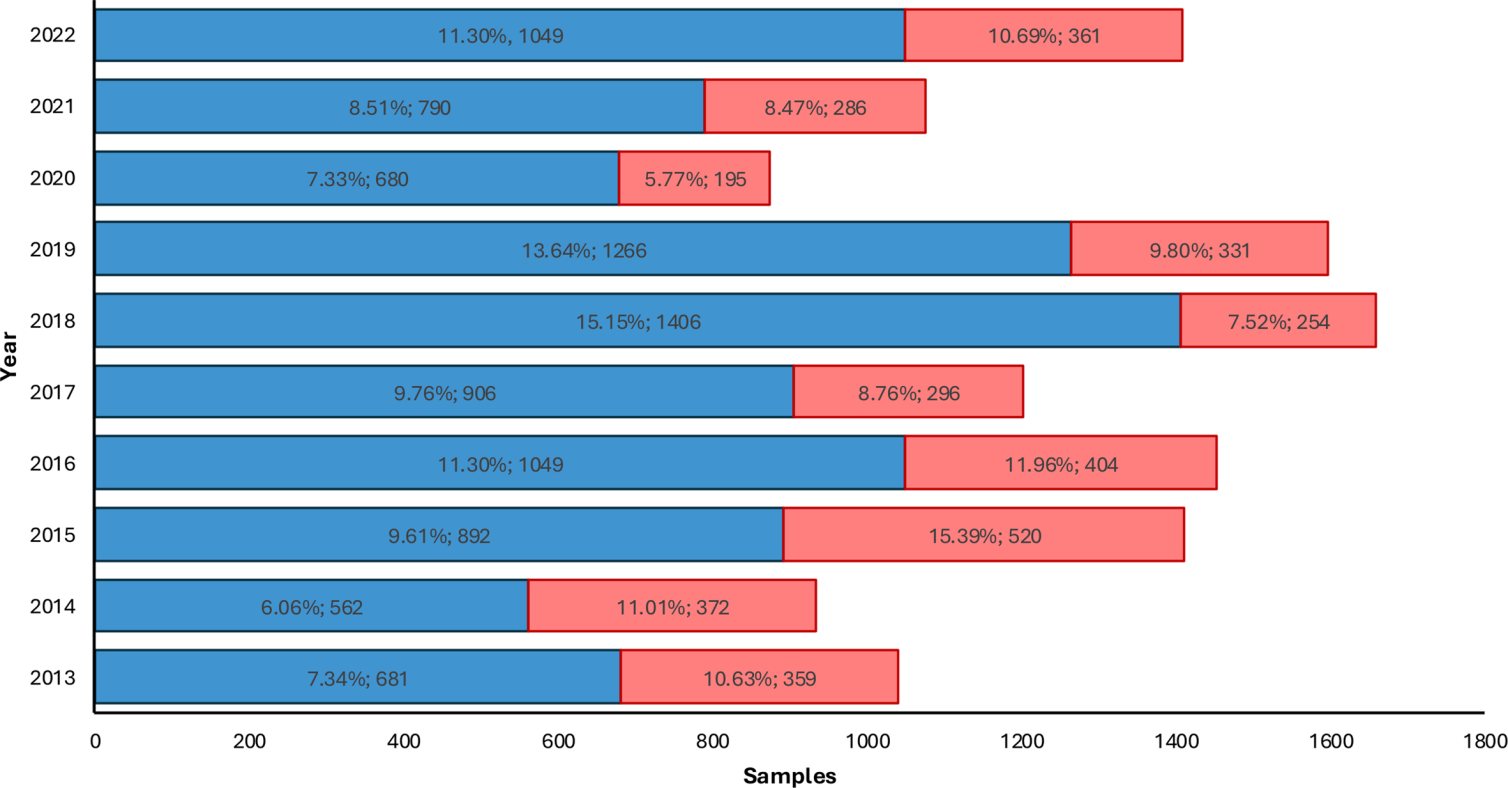
Year‐by‐year distribution (% and number) of hair samples analyzed between 2013 and 2022 from repeated (blue) and chronic (red) alcohol users.

The total number of hair samples (*N* = 68,221), collected between 2013 and 2022, were analyzed by the three drinking categories: does not contradict abstinence (EtG ≤ 5 pg/mg), repeated alcohol consumption (5 < EtG < 30 pg/mg), and chronic excessive alcohol consumption (EtG ≥ 30 pg/mg), according to SoHT criteria. Across the entire observation period, samples not contradicting abstinence consistently represented the majority of tested samples each year (more than 80.8% of the samples), whereas the remaining samples were almost equally divided between chronic (*N* = 3,818, 5.6%) and repeated alcohol consumption (*N* = 9,281, 13.6%). The total number of samples analyzed per year varied substantially over the decade. The sample count appears relatively lower in the first years (e.g., around 4000 samples per year from 2013 to 2014) with an upward trend, reaching higher volumes in later years (e.g., around 7000–8000 samples per year from 2015 to 2022, except for 2020 where they were around 6000), according to the consolidation of hair EtG in assessing chronic abuse. The analysis of chronic (red) and repeated (blue) alcohol users reveals a trend in drinking pattern indicators over the decade (Figure 1). Over the years, repeated alcohol users exceeded chronic ones, indicating a higher prevalence of moderate alcohol intake. In 2020, we observed the markedly lowest prevalence of chronic alcohol drinkers: Hair EtG results from chronic alcohol users accounted for 5.8%.

The samples from non‐abstinent users (*N* = 13,099) were further analyzed by age, gender, applicants, recidivism, sampling region, sampling length, seasonality, and the COVID‐19 pandemic impact.

### 3.2 Age

**FIGURE 2:**
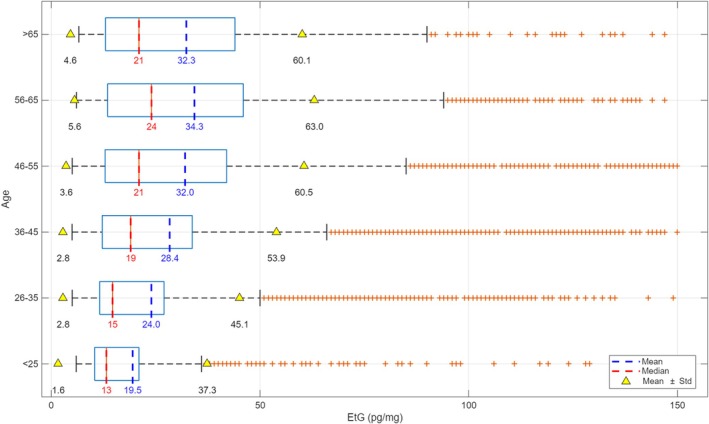
Mean (blue), median (red), and standard deviation (SD, yellow) of EtG concentrations across six age groups (< 25, 26–35, 36–45, 46–55, 56–65, and > 65 years).

Regarding the age of individuals, Figure 2 shows the distribution of mean, median, and standard deviation (SD) of EtG concentrations across six age groups, ranging from under 25 years to over 65 years. A progressive age‐related increase in mean EtG values was observed across the < 25‐ to 65‐years range. The lowest mean concentration was found in individuals under 25 years old (mean: 19.5 pg/mg; median: 13.0 pg/mg), whereas the highest was recorded in the 56–65 age group (mean: 34.3 pg/mg; median: 24.0 pg/mg). However, interindividual variability remained high in all groups, with SDs ranging from 17.8 pg/mg (< 25) to 28.7 pg/mg (56–65). Hair EtG concentrations between age groups were statistically different (*p* = 1.4118 × 10^−69^).

### 3.3 Gender

By gender, women accounted for only a very small proportion (5.8%, *N* = 760) of the samples testing non‐abstinent. Among women, 83.3% (*N* = 633) exhibited EtG concentrations between 5 and 30 pg/mg (repeated consumption), whereas this proportion was 71.1% (*N* = 8,321) among men. Significant differences were observed in terms of EtG concentrations between genders (*p* = 4.0566 × 10^−18^).

### 3.4 Applicants

The overall dataset of subjects tested chronic and repeated alcohol users was also analyzed according to four categories of applicants:

1. Individuals pursuing driver license reinstatement (87.0%).
2. Individuals from recovery services for alcohol abuse (11.2%).
3. Individuals undergoing workplace testing (0.4%).
4. Other (1.4%).

**FIGURE 3:**
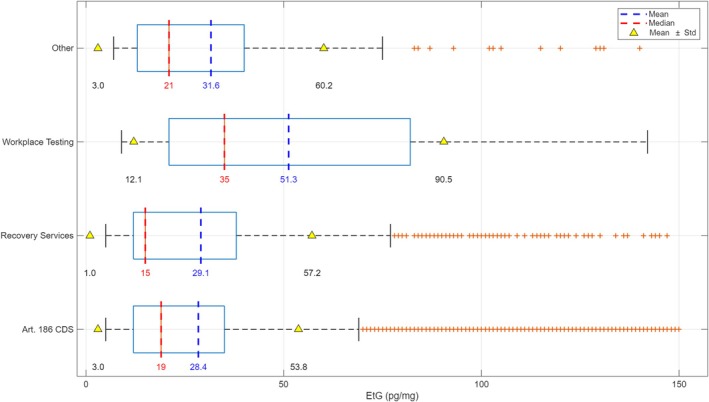
Mean, median, and standard deviation (SD) of EtG concentrations in non‐abstinent samples stratified by referral context: driver license reinstatement (ex‐art. 186 CDS), recovery services for alcohol abuse, workplace testing, and other requests.

The highest mean EtG concentrations were observed in the group of workplace testing (51.3 pg/mg), followed by the group of “other,” category that exhibited the second highest mean EtG concentration (31.6 pg/mg). The recovery services for alcohol abuse showed a mean EtG of 29.1 pg/mg, whereas samples collected as part of driving license reinstatement evaluations (ex‐art. 186 CDS—Codice Della Strada—the Italian Highway code) had the lowest EtG mean concentration (28.4 pg/mg). The one‐way ANOVA yielded a highly significant *p* value (4.4650 × 10^−9^) between groups (Figure 3).

### 3.5 Relapses

**FIGURE 4:**
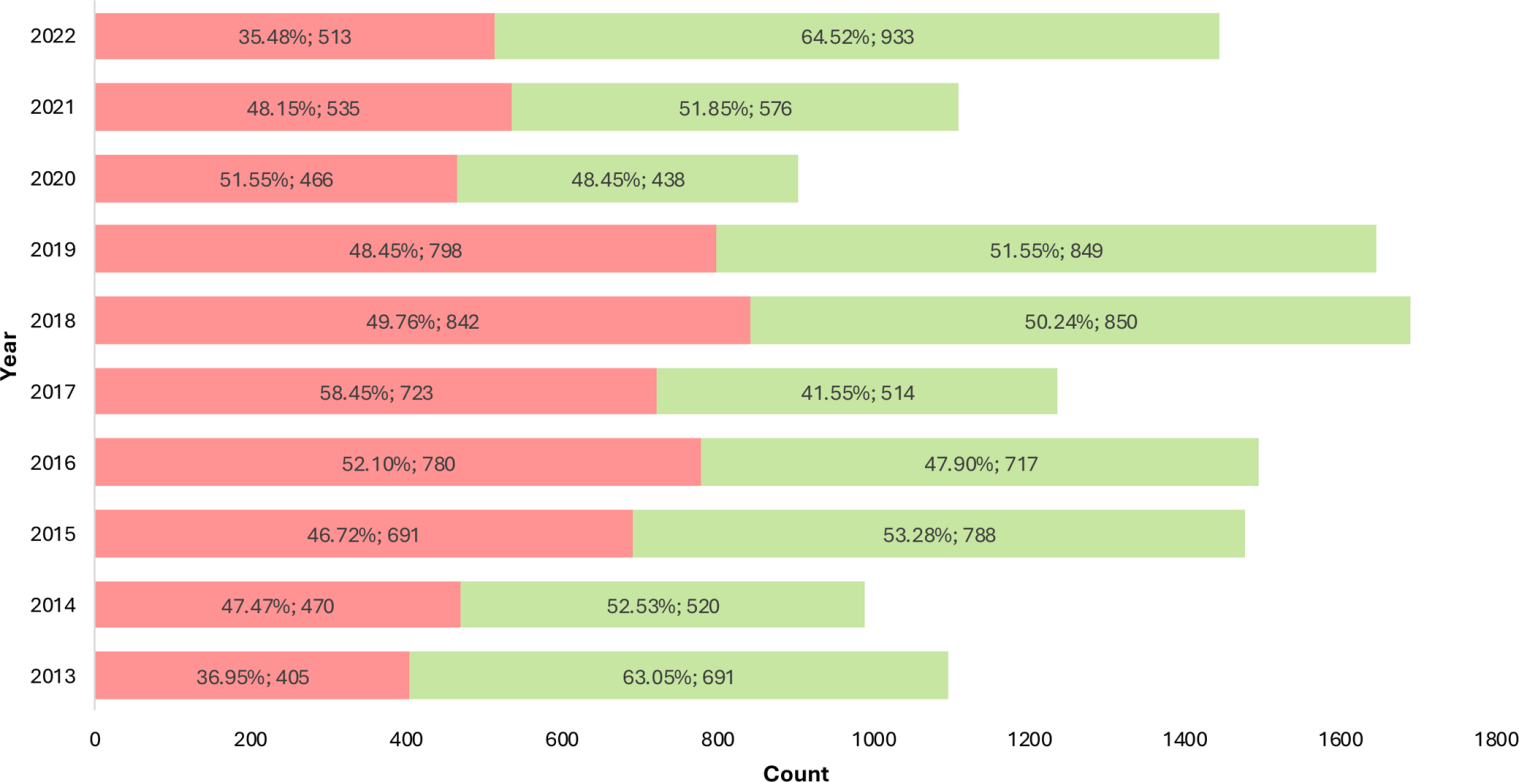
Year‐by‐year distribution of recidivist (red) and nonrecidivist (green) cases from 2013 to 2022.

The year‐by‐year distribution of recidivist (defined as at least two repeated or chronic alcohol consumption results over the considered period) and non‐recidivist cases from 2013 to 2022 is reported in Figure 4, along with their respective counts and percentages within each year. In the early years, non‐recidivist cases appeared to predominate; however, these data cannot be considered reliable, as the use of this alcohol biomarker in hair analysis was first introduced in 2012. In 2016, 2017 and 2020, the proportion of recidivists exceeded that of non‐recidivists, whereas in 2022, we observed the lowest prevalence of recidivists (35.5%).

### 3.6 Sampling Region (Scalp and Body Hair)

**FIGURE 5:**
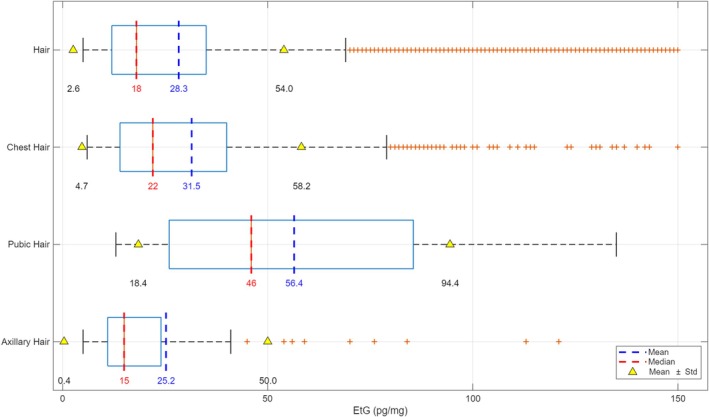
Mean, median, and standard deviation (SD) of EtG concentrations in head hair and body (chest, pubic, and axillary) hair samples.

Because head hair is generally the preferred specimen, almost 91.5% (*N* = 11,987) of the tested hair samples were taken from the head. If head hair is unavailable, other body hair (*N* = 1,114) has been collected: Around 7.4% of samples (*N* = 965) were taken from the chest, and the remaining were pubic (0.7%, *N* = 91) and axillary (0.4%, *N* = 58). The mean, median, and range of EtG concentration in hair and body hair were shown in Figure 5.

In general, the median EtG concentration is considerably lower than the mean, suggesting a right‐skewed distribution with some higher outlier values. The SD range is notably wide, further highlighting the spread of data. The median EtG concentration in head hair (18.0 pg/mg) is quite in accordance with chest hair (22.0 pg/mg), but the ANOVA test stated statistically significant differences between head and chest hair (*p* = 0.0019). Moreover, both distributions are right‐skewed with a significant number of high values, suggesting a high interindividual variability in both hair types. In contrast, axillary hair presented the lowest median concentration of EtG (15.0 pg/mg) and the pubic hair showed the higher one (46.0 pg/mg).

### 3.7 Sampling Length

**FIGURE 6:**
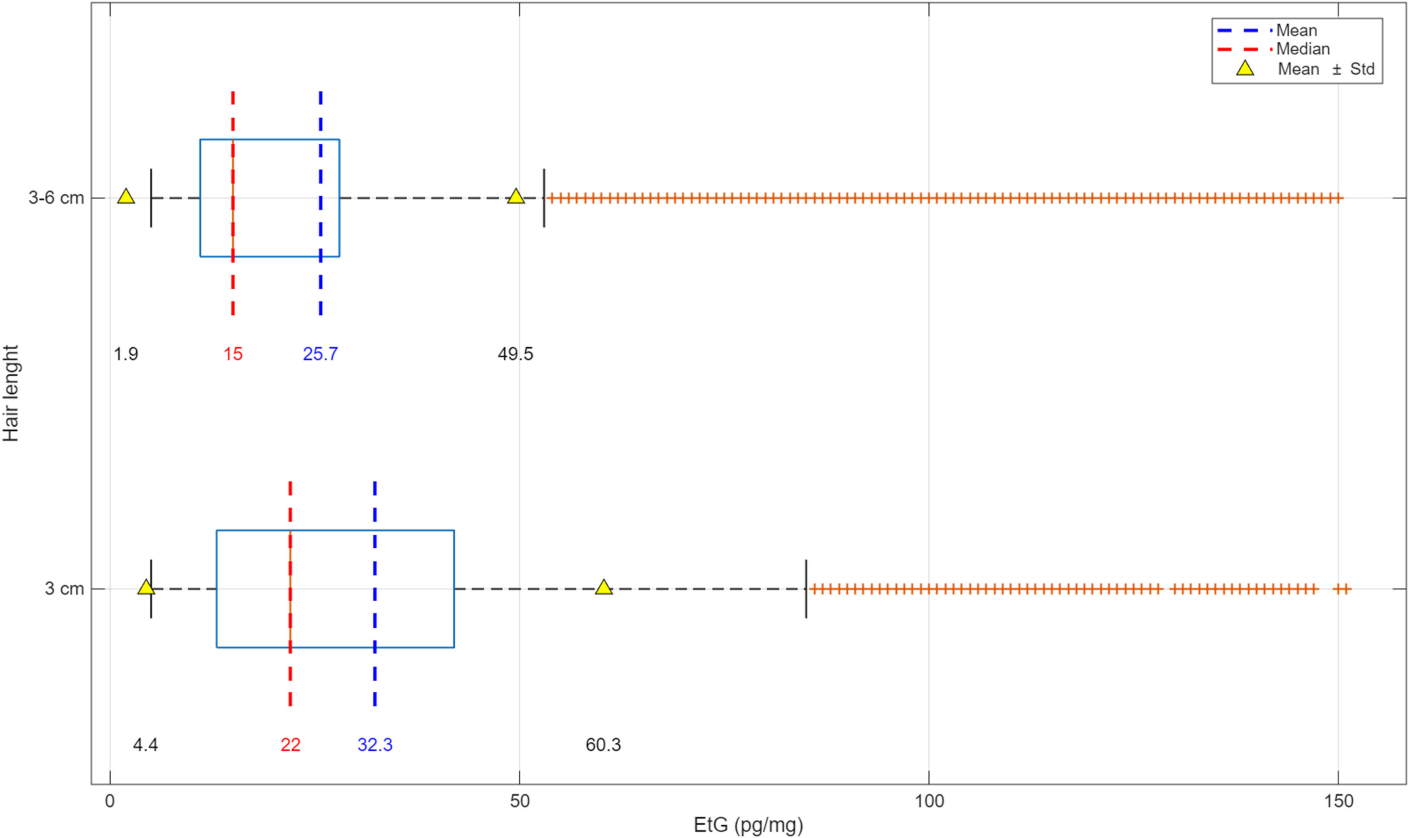
Mean, median, and standard deviation (SD) of EtG concentrations in head hair samples by segment length (3 cm vs. > 3–6 cm).

Concerning head hair, the comparison of sampling lengths (≤ 3 cm vs. > 3–6 cm) exhibit significantly different EtG concentrations (*p* = 1.7307 × 10^−45^). The median and mean hair EtG levels were 22.0 and 32.3 pg/mg for ≤ 3‐cm length and 15.0 and 25.7 pg/mg for > 3‐ to 6‐cm length, respectively. Moreover, analyzing separately, samples with a hair length equal to 3 cm (excluding the samples with a length < 3, *N* = 269, 2%) versus > 3–6 cm, both mean, median, and ANOVA results were comparable (*p* = 2.7353 × 10^−42^) (Figure 6). Moreover, no statistical differences were found between lengths of < 3 cm and equal to 3 cm (*p* = 0.7651).

### 3.8 Seasonality

When head hair EtG from non‐abstinent users was examined across seasons, the one‐way ANOVA resulted in a *p* value of 1.4591 × 10^−14^. Contrarily, body hair showed no significant difference across seasons (*p* = 0.5859).

**FIGURE 7:**
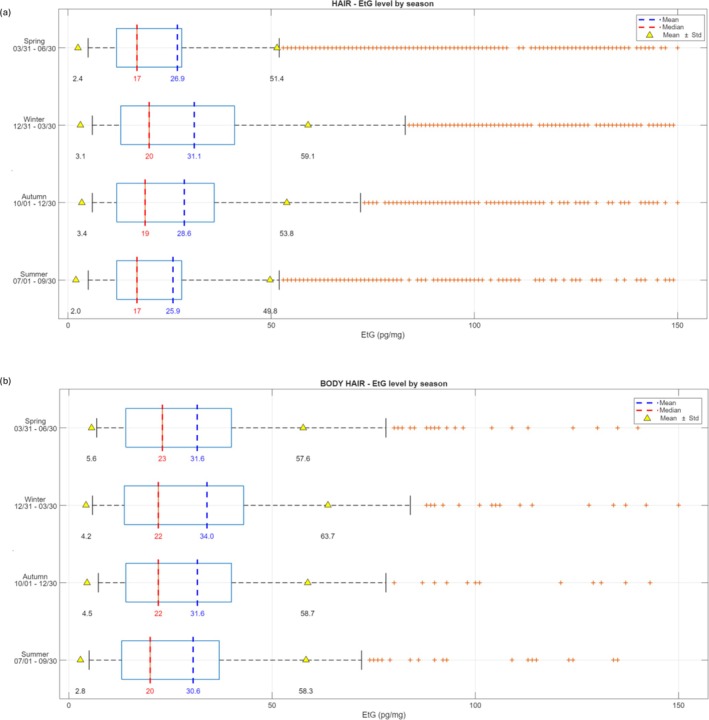
Mean, median, and standard deviation (SD) of scalp hair (above) and body hair (below) samples in the four seasons (month/day).

**FIGURE 8:**
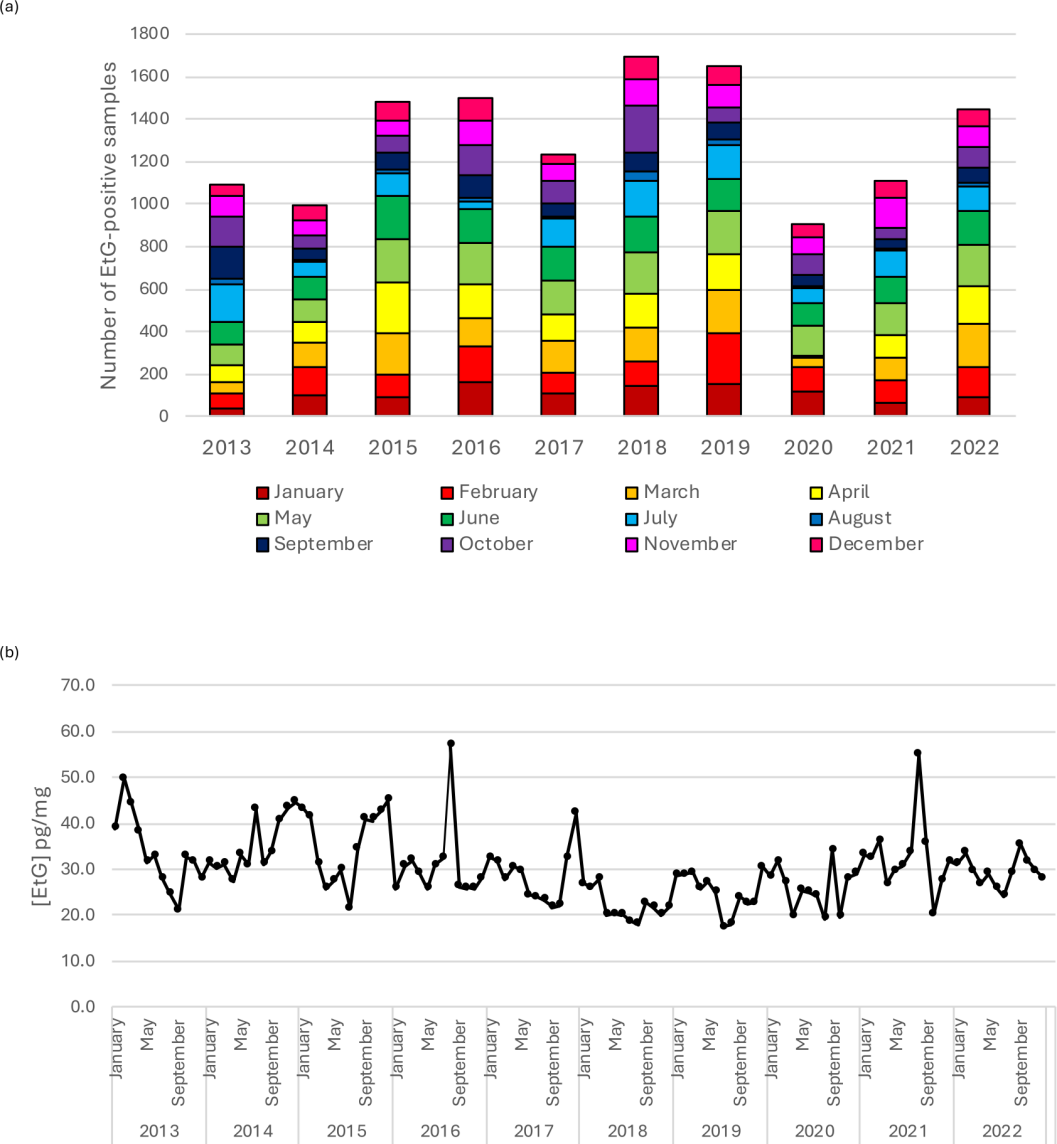
Month‐by‐month number of hair EtG from nonabstinent users analyzed from 2013 to 2022 (a) and time series representing the season‐by‐season median EtG concentration values (b).

In detail, head hair EtG from non‐abstinent users analyzed in winter showed statistically different concentrations in comparison to summer, autumn, and spring (Figure 7), as well as in autumn when compared to summer and spring. No significant differences were highlighted between summer and spring period. The mean and median values for scalp hair samples in the four seasons were, respectively, the following: 25.9 and 17.0 pg/mg in summer, 28.6 and 19 pg/mg in autumn, 31.1 and 20.0 pg/mg in winter, and 26.9 and 17 pg/mg in spring. Comparable values were found for body hair: 30.6 and 20.0 pg/mg in summer, 31.6 and 22.0 pg/mg in autumn, 34.0 and 22.0 pg/mg in winter, and 31.6 and 23.0 pg/mg in spring. The month‐by‐month distribution of hair EtG from non‐abstinent users from 2013 to 2022 and the median EtG concentrations season‐by‐season from 2013 to 2022 were shown in Figure 8.

### 3.9 Impact of COVID‐19 Pandemic

**FIGURE 9:**
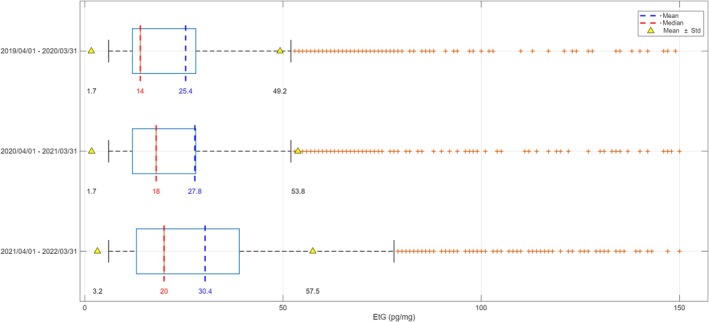
Mean, median, and standard deviation (SD) of EtG concentrations across three distinct time periods: from April 1, 2019, to Mar 1, 2020 (pre‐COVID‐19); from April 1, 2020, to April 1, 2021 (lockdown and immediately post‐COVID‐19); and from April 1, 2021, to April 1, 2022 (post‐COVID‐19 period) (year/month/day).

The influence of the COVID‐19 pandemic on hair EtG results was investigated. The short‐term impact on EtG concentrations was evaluated by comparing the first 4 months (from April 1, 2020, to August 1, 2020) after lockdown measures compared with those reported in the same months during the previous and the post years (2013–2022), as well as the long‐term impact by comparing EtG concentrations across three distinct time periods: (1) from April 1, 2019, to March 1, 2020 (pre‐COVID‐19); (2) from April 1, 2020, to April 1, 2021 (lockdown and immediately post‐COVID‐19); and (3) from April 1, 2021, to Apr 1, 2022 (post‐COVID‐19 period). In the short‐term impact comparisons, no statistical differences in hair EtG concentrations were found (*p* =  0.0980), whereas considering long‐term impact, statistically higher (*p* = 4.3000 × 10^−6^) hair EtG concentrations were only detected in post‐pandemic rather than pre‐pandemic ‐ period (Figure 9).

## 4 Discussion

This 10‐year study of hair EtG concentrations in a large Northern Italian population (*N* = 68,221) provides an extensive overview of chronic alcohol consumption behavior, recidivism, the influence of sampling region, sampling length, and external factors, including seasonality and the COVID‐19 pandemic.

### 4.1 Temporal Trends and Drinking Patterns

Across the decade, samples with EtG concentrations ≤ 5 pg/mg, which do not contradict abstinence according to SoHT criteria, consistently predominated (> 80%). Notably, a clear trend occurred in the distribution of chronic alcohol users (EtG ≥ 30 pg/mg) versus cases indicating repeated alcohol consumption (5 < EtG < 30 pg/mg). Repeated alcohol users predominated in all the considered years, indicating a higher prevalence of moderate *vs*. chronic alcohol intake. This may be indicative of  a consistent pattern of drinking behaviors within the monitored population. In 2020, we observed the markedly lowest prevalence of chronic alcohol users: Hair EtG from repeated alcohol user's results accounted for 5.8% of all non‐abstinent users. Similar reductions in the prevalence of chronic excessive drinking have been reported in other supervised Italian cohorts, suggesting that surveillance and preventive interventions can influence long‐term trends [11].

### 4.2 Demographic Influences

Age‐stratified data revealed a clear progressive increase in mean EtG values with advancing age. This trend suggests that older individuals were more likely to present with higher levels of EtG, potentially reflecting longer term patterns of alcohol consumption or increased likelihood of referral due to chronic use. However, interindividual variability remained high in all groups, with SDs ranging from 17.8 pg/mg (< 25) to 28.7 pg/mg (56–65), indicating substantial individual differences.

Gender‐based analysis confirmed that females accounted for a minority of non‐abstinent users (5.8%), with the majority falling into the repeated alcohol user's category (83.3% for female and 71.1% for male). These findings align with prior reports [11], indicating lower levels of chronic alcohol intake among women subject to legal or occupational monitoring.

### 4.3 Recidivism and Referral Context

In the initial years (2013–2015), non‐recidivist cases predominated, whereas from 2016 onwards recidivist cases became more common, peaking in 2017. However, a resurgence of non‐recidivists was observed in 2022. This fluctuation could be indicative of changes in intervention strategies, public awareness campaigns, or broader socioeconomic factors influencing the likelihood of repeated offenses. Understanding these shifts is crucial for optimizing rehabilitation programs and public health interventions. Furthermore, the study highlighted the impact of the referral context on EtG results. The highest mean EtG concentration in workplace testing samples (51.3 pg/mg) likely reflects policies targeting individuals with known or suspected risky behaviors and underscores the importance of performing such assessment. Samples from recovery services also showed moderate levels (29.1 pg/mg), consistent with the characteristics of the population undergoing treatment. Conversely, driving license reinstatement evaluations had the lowest mean EtG (28.4 pg/mg), possibly indicating less severe or transient issues, or adherence to abstinence. The variation in mean EtG values across categories (driving license reinstatement, recovery services, workplace testing, and others) underscores the importance of considering the requesting entity when interpreting EtG results. The one‐way ANOVA yielded a highly significant *p* value, indicating that the observed differences in EtG levels between these groups are unlikely to be due to random variation alone.

### 4.4 Sampling Site and Length

When comparing EtG concentrations across different hair samples from the same individual, differences may arise from variations in the growth rates and cycles of body hair. Indeed, head hair grows faster (∼1 cm/month) than body hair (< 1 cm/month) and has a considerably longer growth cycle, lasting several years, whereas nonhead hair typically grows for only a few months up to a maximum of 2 years. Thus, when body hair is analyzed, the higher proportion of dormant (telogen) hair should be considered in interpretation [8]. Another possible explanation may be related to differences in exposure: Because urine also contains EtG, it may serve as an additional source of EtG incorporation into pubic hair, potentially leading to higher concentrations [12].

In our study, EtG concentrations were statistically different between scalp (median: 18 pg/mg; mean: 28.3 pg/mg) and chest hair (median: 22 pg/mg; mean: 31.5 pg/mg) (*p* = 0.0019; see Section 3.6). Our results, showing statistically significant differences in EtG concentrations between scalp and chest hair, are consistent with evidence of anatomical variability in EtG incorporation reported in the literature. Pianta et al. [13] and Kerekes et al. [14] both support the use of body hair as an alternative to scalp hair; however, they also highlight that differences in EtG concentrations may reflect both biological variations in incorporation and differences in the time window represented by the matrix. The higher median EtG observed in chest hair may be due to the longer integration period of body hair or qualitative differences in the keratin matrix compared to scalp hair. Therefore, although both matrices are useful for assessing alcohol consumption, our findings emphasize the importance of carefully considering the analyzed hair type, particularly when defining diagnostic cut‐offs or interpreting quantitative alcohol intake in clinical or forensic settings.

Significant differences were highlighted also between head and chest hair versus pubic hair (*p* = 4.6421 × 10^−10^ and *p* = 8.0643 × 10^−8^, respectively), whereas no differences were found among head and chest hair versus axillary hair (*p* = 0.7988 and *p* = 0.2770, respectively). However, as expected, axillary hair presented the lowest median concentration of EtG (15.0 pg/mg), and the pubic hair showed the highest one (46.0 pg/mg). These lower concentrations observed in axillary hair might be probably explained by an increased leaching process due to sweat, as well as higher concentrations in pubic hair might be influenced by urinary or sweaty EtG. Indeed, as published by Kintz et al. [12], pubic hair has showed increased concentrations of EtG in pubic hair (ranging from 12 to 1,370 pg/mg) compared to head hair (< 10 pg/mg). Moreover, Kerekes et al. [15] showed increased EtG concentrations in pubic hair (5–19,647 pg/mg) compared to the concentrations found in head hair (3–1,446 pg/mg).

Regarding the length of head hair sample for EtG analysis, the SoHT consensus [16] initially recommended using the proximal 3 cm of scalp hair; however, the 2011 revision extended the accepted length to the proximal 6 cm, noting that caution is required when analyzing samples shorter than 3 cm [17]. Finally, later editions on alcohol biomarkers also advise caution for samples length less than 3 cm or longer than 6 cm [7]. In our dataset, significant differences were found between both ≤ 3‐cm hair (median 22.0 pg/mg) and equal to 3‐cm hair (median 22.0 pg/mg) versus > 3‐ to 6‐cm hair (median 15.0 pg/mg), suggesting a statistically significant reduction in the distal ends of the hair that could be explained by analyte washout. This finding is consistent with a recent study by Fosen et al. [17], which reported decreasing EtG concentrations in most subjects within a hair segment during growth, when comparing two segments assumed to represent roughly the same period of alcohol intake [18]. Moreover, other previous non‐longitudinal studies have addressed the same question. The study by Agius et al. [19] compared the hair EtG concentrations obtained from 3‐cm hair segments with those from shorter and longer hair segments. They found no significant difference between the hair lengths, suggesting that EtG washout effects play a minor role in the interpretation of EtG results. A study of Tsanaclis et al. [20] determined that normal hair hygiene might wash out EtG from hair. The authors found a significant reduction in EtG values from the first segment to the third segment and concluded that retrospective estimation of alcohol consumption over a period of many months is less useful on routine management of alcohol use. Meier et al. [21] also described decreasing EtG concentrations when comparing segments from seven hair samples taken at different locations on the head (*n* = 1) after a period of constant drinking. Also, in this study, the authors suggested washout effects as a possible explanation of the decreasing EtG concentrations. Conversely, Appenzeller et al. [22] stated that EtG seems to be stable in hair for several months by analyzing EtG concentrations in distal segments (up to 6–8 months old) and alcohol consumption history in 15 patients included in an alcohol treatment program.

Thus, the choice of hair length might introduce a moderate bias in the quantification of alcohol intake, representing an important practical consideration for laboratories and for the interpretation of results.

### 4.5 Seasonality and COVID‐19 Impact

In accordance with the findings of Własiuk et al. [22], who consistently observed seasonal fluctuations in head hair EtG with higher concentrations during colder months and lower values in summer, our dataset also revealed significant seasonal trends (*p* = 1.4591 × 10^−14^), whereas no differences were found in the body hair group (*p* = 0.5859). In detail, hair EtG concentrations in the colder months were significantly higher than those measured during the rest of the year. Thus, differences in the geographic distribution and drinking habits of the study populations did not attenuate or mask seasonal variations in hair EtG levels. These observations suggest that seasonal effects on hair EtG concentrations may not be context or methodology dependent and highlight the importance of considering this variable when interpreting long‐term monitoring data. Several behavioral and environmental factors may explain this bias in hair EtG measurements. For example, higher temperatures can increase perspiration during the warm season, potentially affecting EtG dilution or elimination. The literature [23] also reports a possible washout effect due to prolonged exposure to chlorinated swimming pool water, which may lead to EtG loss. Further targeted studies are needed to determine which of these factors primarily explains the observed seasonal pattern.

Speaking of the impact of the short‐term COVID‐19 pandemic on alcohol consumption patterns, the hair EtG concentration revealed no statistically significant changes (*p =*  0.0980), indicating that, within this monitored population, overall alcohol intake remained consistent despite pandemic‐related restrictions. Contrarily, considering long‐term impact, statistically higher (*p* = 4.3000 × 10^−6^) hair EtG concentrations were detected in the post‐pandemic rather than the pre‐pandemic period.

These findings can be contextualized in light of the study by Alladio et al. [24], which also employed hair EtG as an objective biomarker to assess alcohol consumption during and immediately after the COVID‐19 lockdown in a controlled population in Northern‐Western Italy. In that study, the proportion of individuals classified as abstinent/low‐risk drinkers increased during the lockdown, with a concomitant decrease in moderate and chronic drinkers compared to the previous 4 years, and an overall decrease in mean EtG values in the April–June 2020 period in comparison to 2016–2019, consistent with an immediate reduction in alcohol intake among social drinkers during lockdown conditions. However, Alladio et al. [24] also observed that among chronic/excessive consumers, mean EtG levels tended to increase in June and July 2020, and a pronounced rise was seen for moderate and chronic female drinkers during April–June 2020, suggesting heterogeneity in response by drinking category.

In comparison, our data indicate that in the overall monitored cohort, there were no short‐term statistically significant changes around the lockdown period, whereas the long‐term analysis showed a significant increase in post‐pandemic hair EtG levels compared to pre‐pandemic period. This pattern aligns with the general trend of reduced or stable consumption during COVID‐19 period in the broader population reported by Alladio et al. but partially differs because we did not observe an immediate lockdown‐associated shift. Consistent with Alladio et al.'s observation of increased EtG among certain subgroups of heavy drinkers following lockdown, our data also support a long‐term EtG increase suggesting a pandemic‐related modification of drinking behavior.

In a longitudinal cohort study using smart‐breathalyzer data, Huston et al. [25] revealed a decrease in drinking between January 1, 2020, and March 30, 2020; an increase between March 30, 2020, and May 25, 2020; a statistically insignificant decrease between May 25, 2020, and January 1, 2021; and an increase again between January 1, 2021, and June 4, 2021; however, no statistically significant relationships between shelter‐in‐place orders and alcohol consumption were detected, suggesting the complex relationship between the pandemic and alcohol consumption patterns.

As highlighted by the review article by Merlo et al. [26], which analyzed more than 100 studies worldwide, overall findings indicated no change (51%) or a reduction (23%) in alcohol consumption during the COVID‐19 pandemic. However, across countries, on average one in four individuals reported an increase in alcohol consumption (26%), in particular during the COVID‐19 lockdown periods. Most common correlates of increased alcohol consumption were being female, having a child at home, higher educational level, and poorer mental health (including higher scores for stress, anxiety, and depression). Our results are therefore consistent with the global evidence summarized by Merlo et al. [26], reinforcing the view that although specific subgroups may have increased their alcohol intake during the lockdown, population‐level monitoring through hair EtG does not indicate a substantial overall rise in consumption. This alignment suggests that the pandemic's impact on drinking was heterogeneous and context dependent. 

### 4.6 Strengths and Limitations

Strengths of this study include its large sample size, decade‐long observation period, and inclusion of diverse forensic, clinical, and occupational populations. The use of SoHT‐recommended cut‐off of 30 pg/mg for chronic alcohol consumption ensures international comparability. Limitations include potential selection bias toward individuals under legal or occupational supervision, lack of detailed self‐reported consumption data and cosmetic treatments, and inability to assess short‐term episodic drinking beyond the analyzed hair segment.

## 5 Conclusions

Overall, this comprehensive 10‐year dataset provides robust evidence for the performance of hair EtG as a long‐term biomarker of alcohol intake and reveals nuanced trends in chronic consumption and recidivism within a Northern Italian population. The findings highlight the importance of methodological factors, such as sampling site and length, as well as contextual influences, such as the seasonal variation, in the interpretation of EtG results. In contrast, our data indicate that in the overall monitored cohort, there were no short‐term statistically significant changes around the lockdown period, whereas the long‐term analysis showed a significant increase in post‐pandemic hair EtG levels relative to pre‐pandemic period. However, the substantial interindividual variability of hair EtG concentration indicates that any general surveillance policy should be applied with appropriate caution.

## Funding

This study was partially funded by the Italian Ministry of Heath (Ministero della Salute)—Current Research (RC25) Fondazione IRCSS Ca' Granda Ospedale Maggiore Policlinico.

## Conflicts of Interest

The authors declare no conflicts of interest.

## Data Availability

The data that support the findings of this study are available on request from the corresponding author.
